# Microbial community associated with the crustose lichen *Rhizocarpon geographicum* L. (DC.) living on oceanic seashore: A large source of diversity revealed by using multiple isolation methods

**DOI:** 10.1111/1758-2229.13105

**Published:** 2022-07-20

**Authors:** Alice Miral, Patricia Jargeat, Lengo Mambu, Isabelle Rouaud, Sylvain Tranchimand, Sophie Tomasi

**Affiliations:** ^1^ Univ Rennes, CNRS, ISCR (Institut des Sciences Chimiques de Rennes)‐UMR 6226 Rennes France; ^2^ UMR 5174 UPS‐CNRS‐IRD Laboratoire Evolution et Diversité Biologique, EDB Université Toulouse‐3, Bât 4R1 Toulouse France; ^3^ EA 7500 Laboratoire PEIRENE, Faculté de Pharmacie Université de Limoges Limoges Cedex France; ^4^ Ecole Nationale Supérieure de Chimie de Rennes, CNRS, ISCR (Institut des Sciences Chimiques de Rennes)‐UMR 6226 Université de Rennes Rennes France

## Abstract

Recently, the study of the interactions within a microcosm between hosts and their associated microbial communities drew an unprecedented interest arising from the holobiont concept. Lichens, a symbiotic association between a fungus and an alga, are redefined as complex ecosystems considering the tremendous array of associated microorganisms that satisfy this concept. The present study focuses on the diversity of the microbiota associated with the seashore located lichen *Rhizocarpon geographicum*, recovered by different culture‐dependent methods. Samples harvested from two sites allowed the isolation and the molecular identification of 68 fungal isolates distributed in 43 phylogenetic groups, 15 bacterial isolates distributed in five taxonomic groups and three microalgae belonging to two species. Moreover, for 12 fungal isolates belonging to 10 different taxa, the genus was not described in GenBank. These fungal species have never been sequenced or described and therefore non‐studied. All these findings highlight the novel and high diversity of the microflora associated with *R*. *geographicum*. While many species disappear every day, this work suggests that coastal and wild environments still contain an unrevealed variety to offer and that lichens constitute a great reservoir of new microbial taxa which can be recovered by multiplying the culture‐dependent techniques.

## INTRODUCTION

Coastal environments and cliffs, whether wave‐pounded or inland, are binding ecosystems but are also very fragile. They occupy one of the most dynamic interfaces on Earth, at the boundary between land and sea and finally correspond to some of the most diverse and productive habitats (McLean et al., 2001). Considered as climatic refugia, they arouse growing scientific interest. Their ecology and biogeography have been investigated across the world (Kuntz & Larson, 2006; Strumia et al., 2020). As a brittle environment, cliffs and seashores are not only naturally unstable and subject to rapid changes (exposure to waves, local marine currents and wind action), but they are also affected by global climate change (rise of temperature, changes in precipitation regimes, water acidification, sea level, wave exposure and salt spray) modifying the physical, biological and biogeochemical characteristics of the oceans and coasts as well as their ecological structure and functions. Due to such factors, several cliff plant species and their underexplored communities are under the threat of extinction, requiring political action for their conservation. The Integrated Coastal Zone Management and the European Biodiversity Strategy are examples of spatial planning strategies such as the protection by Council Directive 92/43/EEC (European Economic Community) (Pena et al., 2021; Strumia et al., 2020).

Coastal plant communities grow under specific environmental conditions explained by the interaction of land and sea, and lichens are an essential component of such communities. According to the classic definition, lichens are symbiotic organisms, highly adapted to extreme habitats (Sancho et al., 2007), formed with a fungal partner, the mycobiont, and a photoautotrophic partner, the photobiont.

With its attractive green‐yellow colour, *Rhizocarpon geographicum* (L.) DC. (Rhizocarpaceae, Ascomycota) (McCarthy & Elix, 2014), formed by the fungi *Rhizocarpon* (Ascomycota, Lecanoromycetes, Rhizocarpaceae) and the microalga *Trebouxia* sp. (Chlorophyta, Trebouxiophyceae), is one of the most widely distributed crustose lichens and frequently one of the first colonizers of newly exposed rock surfaces, first terrestrial substrates available for living organisms on Earth (Ruibal et al., 2009). This species grows exceptionally slowly, on a broad range of substrates, occasionally found in submontane regions, but more commonly in the high mountains (Armstrong, 2011), and is thereby a difficult subject for laboratory conditions (Armstrong & Smith, 1996). As other rock‐inhabiting lichens, it is often exposed to extreme abiotic conditions with broad fluctuations of temperature and humidity providing poor comfort of life and sources of nutrients (Muggia & Grube, 2018), conferring them unique abilities to develop protective mechanisms (Fernandes et al., 2015). As crustose lichens are found in high altitude habitat, the samples studied in this work, collected at La Pointe de Crozon, Brittany (France), are strongly affected by hostile environmental conditions. As for lichens of the arctomontane group, which is associated with the Arctic and high mountain regions, their presence is probably explained by severe environmental conditions in coastal habitats (Rodnikova, 2012).

Few years ago, it was admitted that a third partner, the microbial consortia of bacteria and fungi, was part of the evolutionary, long‐term successful and intimate lichen lifestyle (Grimm et al., 2021; Grube et al., 2009; Spribille et al., 2016). Nowadays, lichens are considered as holobionts (Simon et al., 2019), an exciting reservoir and unexplored hotspot of more or less specific and persistent members of complex microbial networks (Cao et al., 2018; Cardinale et al., 2006). However, their role in adapting lichens to unfriendly environments and moreover to coastal environments is still not clear (Delmail et al., 2013). If these microbial networks are dependent on these particular habitats, they might be involved in defence and chemical communication pathways as a source of original molecules (Bjelland et al., 2011; Boustie et al., 2011; Suzuki et al., 2016). The diversity and contribution of this third partner, the fungal and bacterial consortia partner, have recently been studied mostly by culture‐independent techniques (Cardinale et al., 2008; Muggia & Grube, 2018) due to difficulties in their isolation and uncultivability. They have been described as the ‘microbial dark matter’ (Rinke et al., 2013). However, these techniques can lead to biased estimates of microbial community richness and composition. In order to offset molecular analysis' lack of information and hardship of high recovering percentage of cultivable strains in axenic cultures, numerous media and different techniques of isolation can be applied (Lagarde et al., 2018; Muggia et al., 2017).

Most of the studies reported the influence of the culture media on the composition and diversity of the isolated microorganisms (Li & Wang, 2017; Medina et al., 2017; Muggia et al., 2017) and that this composition is also affected by the employed method of lichen's surface sterilization (Masumoto & Degawa, 2019). In this study, we aimed to investigate the microbial community of two samples of the lichen *R*. *geographicum* collected in the westernmost and hostile point of France, facing the Atlantic Ocean, in order to explore the French local biodiversity by depicting the common lichen‐associated microbiota but also to improve our microbial diversity knowledge by reporting non‐described and non‐studied microbial strains.

## EXPERIMENTAL PROCEDURES

### Lichen sample collection

The two thallus samples of *R*. *geographicum* were carefully collected in February 2020, under specific municipality authorization, using sterile gloves and washed instruments, in France at La Pointe de Crozon from two different locations: site 1 (48°13′59″N and 4°33′60″W) and site 2 (48°14′11″N and 4°33′59″W) located in a very particular spot: on a seaside cliff and directly exposed to the ocean sprays. Lichen samples were identified by Joel Esnault from the French Association of Lichenology.

After sampling, lichens on rock fragments were transported in sterile Petri dishes stored in individual plastic bags and processed within 6 hours.

### Isolation and culture conditions

As there is no standardized methodology for the isolation of lichen‐associated microbiota, a protocol for lichen washing was used, based on Parrot et al. (2015) and Petrini (1991). Two techniques of isolation and 11 different media picked on DMSZ website (https://www.dsmz.de/collection/catalogue/microorganisms/culture‐technology/list‐of‐media‐for‐microorganisms) were applied: Glucose Yeast Extract Medium (GEM) containing dextrose (20 g L^−1^); yeast extract (10 g L^−1^), CaCO_3_ (20 g L^−1^), agar (15 g L^−1^); Malt Extract Peptone Agar (MEP): malt extract (30 g L^−1^), soya peptone (3 g L^−1^), agar (15 g L^−1^); Potato Dextrose Agar (PDA): potato extract (4 g L^−1^), dextrose (20 g L^−1^), agar (15 g L^−1^); Luria–Bertani Agar (LB): tryptone (10 g L^−1^), yeast extract (5 g L^−1^), sodium chloride (0.5 g L^−1^), agar (15 g L^−1^); Tryptone Yeast Extract Medium modified Agar (TY): tryptone (10 g L^−1^), yeast extract (5 g L^−1^), sodium chloride (5 g L^−1^), agar (15 g L^−1^); Peptone Yeast Extract Medium with MgSO_4_ Agar (PYM): peptone (10 g L^−1^, yeast extract (1 g L^−1^), MgSO_4_·7 H_2_O (2 g L^−1^), (NH_4_)_2_SO_4_ (2 g L^−1^), agar (15 g L^−1^); Yeast Starch Agar (YS): yeast extract (2 g L^−1^), soluble starch (10 g L^−1^), agar (15 g L^−1^); Mannitol Yeast Extract Peptone (MYP): d‐mannitol (25 g L^−1^), yeast extract (5 g L^−1^), peptone (3 g L^−1^), agar (15 g L^−1^). Marine Agar (MB) bacto peptone (5 g L^−1^); bacto yeast extract (1 g L^−1^); agar (15 g L^−1^), Gym Streptomyces Agar (GYM): dextrose (4 g L^−1^), yeast extract (4 g L^−1^), malt extract (10 g L^−1^), CaCO_3_ (2 g L^−1^), agar (15 g L^−1^); and ISP2 (ISP2): dextrose (4 g L^−1^), yeast extract (4 g L^−1^), malt extract (10 g L^−1^), agar (15 g L^−1^). Aseptically, the crustose lichen *R*. *geographicum* was scrapped from the rock using a sterile scalpel and lichen sample obtained was split into two sterile 50 ml Falcon® tubes. 20 ml of sterile distilled water was added to the first tube and a 1 min vortexing was applied. After decantation, the supernatant was removed and the washing was carried out two more times. The third washing water was kept and used for isolation. 200 μL of this supernatant was spread plated on 11 different media and incubated at room temperature until growth of fungi and bacteria. The second part of thalli was washed as described above, and then transferred into an empty Petri plate to dry in the laminar flow hood. Four little fragments (1 × 1 mm) of thalli were then deposited on the 11 different media previously described, in Petri dishes, and incubated at room temperature until growth of fungi and bacteria. A total of 88 thallus fragments were incubated. For each plate, the isolates and colonies were daily examined for 4 days to 6 months. The ones showing distinct phenotypes were transferred into new Petri dishes containing the respective medium until pure culture. All pure isolates were stored at −80°C in cryotubes containing 20% sterile glycerol.

### Morphology‐based comparisons

Identification of fungi was carried out by examination of the fungal fruiting bodies and the mycelia under a microscope (Olympus CX41) after the growth of the fungi on PDA in Petri dishes to obtain fungal colonies. After 8–15 days at room temperature, morphological identification was performed based on the colours and shapes of the colonies, as also performed for bacteria and microalgae.

### Molecular identifications

For the molecular identification of fungal isolates, two protocols were used. The protocol of DNA extraction and ITS PCR amplification used at EDB lab (Toulouse, France) was previously described (Lagarde et al., 2018). For identification at the species level of the *Penicillium* isolates, beta‐tubulin and calmodulin regions were PCR‐amplified using the primers Bt2a/Bt2b (Glass & Donaldson, 1995) and CMD5/CMD6 (Hong et al., 2005), in the same conditions as above, except for annealing temperature (58°C instead of 55°C). PCR products were sequenced by Eurofins Genomics (Ebersberg, Germany) using ITS 5, Bt2a and CMD5 primers, respectively.

Regarding the protocol used at the Bio2Mar platform (Banyuls sur Mer, France), total DNA was extracted from mycelia directly picked from the Petri dishes and then transferred onto FTA® paper, using the Whatman FTA Protocol BD05. The disc was then placed in a PCR amplification tube (1.5 mL microcentrifuge tube) and 200 μL of FTA Purification Reagent was added. The amplification tube was incubated for 5 min at room temperature with moderate manual mixing. The Purification Reagent was then removed and discarded with a pipette. The three later steps were repeated once for a total of two washes. 200 μL of TE^−1^ buffer (10 mM Tris–HCl, 0.1 mM EDTA, pH 8.0) was added and the mix was incubated for 5 min at room temperature. The disc was removed and two more TE^−1^ Buffer washes were performed prior to the analysis. The ITS rDNA region was PCR‐amplified using the primer set ITS1/ITS4 (White et al., 1991) and the 18S rDNA gene was PCR‐amplified using the oligonucleotide primers 25F (5′‐ACCTGGTTGATCCTGCCAG‐3′) and 1515R (5′‐TGATCCTTCYGCAGGTTCAC‐3′). Molecular bacterial and microalgal identification was carried out according to Fagervold et al. (2013) and Hadi et al. (2016).

Sequence data are available in GenBank under accession numbers OLO891597 and OL891600 to OL891636 (fungal ITS), OL890684 to OL890687 (18S), OL891637 to OL891641 (bacterial 16S) and OL891598 to OL89599 (algal ITS) (Tables 1, 2, 3). Only one sequence per taxa was deposited.

**TABLE 1:** Molecular and morphological identification of fungi isolated from *R*. *geographicum*

				Classification				
Isolate	BLAST and Phylogenetic identification	Location	Deposit method	Phylum	Class	Order	Family	Accession number
B01	Undet. Cystobasidiomycetes^a^	Site 1	Supernatant	Basidiomycota	Cystobasidiomycetes	*Incertae sedis*		OL890684
B03	*Phaeosclera* sp.	Site 2	Supernatant	Ascomycota	Dothideomycetes	*Incertae sedis*		OL891597
B05a	*Aureobasidium* sp.^a^	Site 2	Thallus	Ascomycota	Dothideomycetes	Dothideales	Dothioraceae	OL890685
B08a	*Filobasidium uniguttulatum*	Site 2	Supernatant	Basidiomycota	Tremellomycetes	Filobasidiales	Filobasidiaceae	OL891601
B24a	*Tremella* sp.^a^	Site 1	Thallus	Basidiomycota	Tremellomycetes	Tremellales	Tremellaceae	OL890686
B27	Undet. Myriangiales^a^	Site 2	Supernatant	Ascomycota	Dothideomycetes	Myriangiales	N.D.	OL890687
B28	*Thyronectria sinopica*	Site 2	Thallus	Ascomycota	Sordariomycetes	Hypocreales	Nectriaceae	OL891600
C01	*Coprinellus disseminatus*	Site 2	Thallus	Basidiomycota	Agaricomycetes	Agaricales	Psathyrellaceae	OL891602
C02	*Xylaria hypoxylon*	Site 2	Thallus	Ascomycota	Sordariomycetes	Xylariales	Xylariaceae	OL891603
C03	Undet. Xylariales	Site 2	Supernatant	Ascomycota	Sordariomycetes	Xylariales	N.D.	OL891604
C04	*Dendrothyrium variisporum*	Site 1	Thallus	Ascomycota	Dothideomycetes	Pleosporales	Didymosphaeriaceae	OL891605
C05	*Xylaria hypoxylon* ^b^	Site 1	Thallus	Ascomycota	Sordariomycetes	Xylariales	Xylariaceae	
C06	*Leptosphaeria rubefaciens*	Site 1	Thallus	Ascomycota	Dothideomycetes	Pleosporales	Leptosphaeriaceae	OL891606
C07	*Hypoxylon petriniae*	Site 1	Thallus	Ascomycota	Sordariomycetes	Xylariales	Xylariaceae	OL891607
C08	*Didymella* sp.	Site 1	Thallus	Ascomycota	Dothideomycetes	Pleosporales	Didymellaceae	OL891608
C09	*Coccinonectria rusci*	Site 2	Thallus	Ascomycota	Sordariomycetes	Hypocreales	Nectriaceae	OL891609
C10a	*Didymocyrtis brachylaenae*	Site 2	Thallus	Ascomycota	Dothideomycetes	Pleosporales	Phaeosphaeriaceae	OL891610
C10b	*Phlebia rufa*	Site 2	Thallus	Basidiomycota	Agaricomycetes	Polyporales	Meruliaceae	OL891611
C11	*Melanconium hedericola*	Site 2	Thallus	Ascomycota	Sordariomycetes	Diaporthales	Melanconidaceae	OL891612
C12	*Didymosphaeria variabile*	Site 2	Thallus	Ascomycota	Dothideomycetes	Pleosporales	Didymosphaeriaceae	OL891613
C13	Undet. Didymosphaeriaceae	Site 2	Thallus	Ascomycota	Dothideomycetes	Pleosporales	Didymosphaeriaceae	OL891614
C14	*Tolypocladium* sp. *(2)*	Site 1	Thallus	Ascomycota	Sordariomycetes	Hypocreales	Ophiocordycipitaceae	OL891615
C15	*Phaeosphaeria* sp.	Site 2	Thallus	Ascomycota	Dothideomycetes	Pleosporales	Phaeosphaeriaceae	OL891616
C16a	*Stemphylium vesicarium*	Site 2	Thallus	Ascomycota	Dothideomycetes	Pleosporales	Pleosporaceae	OL891617
C16b	*Cladosporium* sp.	Site 2	Thallus	Ascomycota	Dothideomycetes	Cladosporiales	Cladosporiaceae	OL891618
C17	*Penicillium roseoviride*	Site 2	Thallus	Ascomycota	Eurotiomycetes	Eurotiales	Aspergillaceae	OL891619
C18	Undet. Phaeosphaeriaceae	Site 2	Thallus	Ascomycota	Dothideomycetes	Pleosporales	Phaeosphaeriaceae	OL891620
C19	*Didymosphaeria variabile*	Site 2	Thallus	Ascomycota	Dothideomycetes	Pleosporales	Didymosphaeriaceae	
C20	Undet. Pleosporales (1)	Site 2	Thallus	Ascomycota	Dothideomycetes	Pleosporales	N.D.	OL891621
C21	Undet. Pleosporales (2)	Site 2	Thallus	Ascomycota	Dothideomycetes	Pleosporales	N.D.	OL891622
C22	*Penicillium cavernicola*	Site 2	Thallus	Ascomycota	Eurotiomycetes	Eurotiales	Aspergillaceae	OL891623
C23	*Trichoderma* sp. *(1)*	Site 1	Thallus	Ascomycota	Sordariomycetes	Hypocreales	Hypocreaceae	OL891624
C24	*Beauveria malawiensis* ^b^	Site 2	Thallus	Ascomycota	Sordariomycetes	Hypocreales	Cordycipitaceae	
C25	*Beauveria malawiensis*	Site 2	Thallus	Ascomycota	Sordariomycetes	Hypocreales	Cordycipitaceae	OL891625
C26	*Stemphylium vesicarium* ^b^	Site 2	Thallus	Ascomycota	Dothideomycetes	Pleosporales	Pleosporaceae	
C27	*Stemphylium vesicarium*	Site 2	Thallus	Ascomycota	Dothideomycetes	Pleosporales	Pleosporaceae	
C28	*Hypoxylon howeanum*	Site 1	Thallus	Ascomycota	Sordariomycetes	Xylariales	Xylariaceae	OL891626
C29	*Hypoxylon howeanum*	Site 2	Thallus	Ascomycota	Sordariomycetes	Xylariales	Xylariaceae	
C30	*Tolypocladium* sp. *(1)*	Site 1	Thallus	Ascomycota	Sordariomycetes	Hypocreales	Ophiocordycipitaceae	OL891627
C31	*Penicillium scabrosum*	Site 1	Thallus	Ascomycota	Eurotiomycetes	Eurotiales	Aspergillaceae	OL891628
C32	*Cytospora* sp.	Site 1	Supernatant	Ascomycota	Sordariomycetes	Hypocreales	Cytosporaceae	OL891629
C33	Undet. Pleosporales (2)	Site 2	Thallus	Ascomycota	Dothideomycetes	Pleosporales	N.D.	
C34	*Stemphylium vesicarium*	Site 2	Thallus	Ascomycota	Dothideomycetes	Pleosporales	Pleosporaceae	
C35	*Coprinellus micaceus*	Site 2	Thallus	Basidiomycota	Agaricomycetes	Agaricales	Psathyrellaceae	OL891630
C36	Undet. Didymosphaeriaceae	Site 2	Thallus	Ascomycota	Dothideomycetes	Pleosporales	Didymosphaeriaceae	
C37	*Stemphylium vesicarium*	Site 2	Thallus	Ascomycota	Dothideomycetes	Pleosporales	Pleosporaceae	
C38	*Stemphylium vesicarium* ^b^	Site 2	Thallus	Ascomycota	Dothideomycetes	Pleosporales	Pleosporaceae	
C39	*Coniothyrium dispersellum*	Site 2	Thallus	Ascomycota	Dothideomycetes	Pleosporales	Leptosphaeriaceae	OL891631
C40	Undet. Pleosporales (2)	Site 2	Thallus	Ascomycota	Dothideomycetes	Pleosporales	N.D.	
C41	*Dendrothyrium variisporum* ^b^	Site 1	Thallus	Ascomycota	Dothideomycetes	Pleosporales	Didymosphaeriaceae	
C42	*Didymosphaeria variabile*	Site 1	Thallus	Ascomycota	Dothideomycetes	Pleosporales	Didymosphaeriaceae	
C43	*Tolypocladium* sp. *(2)*	Site 1	Thallus	Ascomycota	Sordariomycetes	Hypocreales	Ophiocordycipitaceae	
C44	*Microdochium phragmitis*	Site 1	Thallus	Ascomycota	Sordariomycetes	Xylariales	Microdochiaceae	OL891632
C45	*Tolypocladium* sp. *(2)*	Site 1	Thallus	Ascomycota	Sordariomycetes	Hypocreales	Ophiocordycipitaceae	
C46	*Trichoderma* sp. *(1)*	Site 2	Supernatant	Ascomycota	Sordariomycetes	Hypocreales	Hypocreaceae	
C47	*Botrytis cinerea*	Site 2	Thallus	Ascomycota	Leotiomycetes	Helotiales	Sclerotiniaceae	OL891633
C48	*Botrytis cinerea* ^†^	Site 2	Thallus	Ascomycota	Leotiomycetes	Helotiales	Sclerotiniaceae	
C49	*Alternaria* sp. *(2)*	Site 2	Thallus	Ascomycota	Dothideomycetes	Pleosporales	Pleosporaceae	OL891634
C50	*Penicillium scabrosum*	Site 1	Thallus	Ascomycota	Eurotiomycetes	Eurotiales	Aspergillaceae	
C51	*Tolypocladium* sp. *(1)*.	Site 1	Thallus	Ascomycota	Sordariomycetes	Hypocreales	Ophiocordycipitaceae	
C52	*Alternaria* sp. *(1)*	Site 1	Thallus	Ascomycota	Dothideomycetes	Pleosporales	Pleosporaceae	
C55	*Tolypocladium* sp. *(1)*	Site 1	Thallus	Ascomycota	Sordariomycetes	Hypocreales	Ophiocordycipitaceae	
C60	*Tolypocladium* sp. *(1)*	Site 1	Supernatant	Ascomycota	Sordariomycetes	Hypocreales	Ophiocordycipitaceae	
C61	Undet. Chaetothyriales	Site 1	Supernatant	Ascomycota	Eurotiomycetes	Chaetothyriales	N.D.	OL891635
C63a	*Alternaria* sp. *(2)*	Site 2	Thallus	Ascomycota	Dothideomycetes	Pleosporales	Pleosporaceae	
C63b	*Penicillium roseoviride*	Site 2	Thallus	Ascomycota	Eurotiomycetes	Eurotiales	Aspergillaceae	
C66	Undet. *Dothideomycetes*	Site 2	Supernatant	Ascomycota	Dothideomycetes	N.D.	N.D.	OL891636
C72	*Leptosphaeria rubefaciens*	Site 1	Thallus	Ascomycota	Dothideomycetes	Pleosporales	Leptosphaeriaceae	

^a^ 18S sequence.

^b^ Morphological identification, N.D.: not determined.

**TABLE 2:** Bacteria isolated from *R*. *geographicum*

					Classification				
Isolates	BLAST and Phylogenetic identification	Location	Deposit method	BLAST result	Phylum	Class	Order	Family	Accession number
B02	*Lichenibacterium* sp.	Site 1	Supernatant	98% *Lichenibacterium ramalinae*	Proteobacteria	α‐proteobacteria	Hyphomicrobiales	Lichenibacteriaceae	
B04	Undet	Site 2	Supernatant	95.4% *Arthrobacter* spp.	Actinobacteria	Actinomycetia	Micrococcales	Micrococcaceae	OL891637
B05b	*Paenibacillus etheri*	Site 2	Thallus	99.8% *Paenibacillus etheri*	Firmicutes	Bacilli	Bacillales	Paenibacillaceae	
B07	*Paenibacillus etheri*	Site 2	Supernatant	99.8% *Paenibacillus etheri*	Firmicutes	Bacilli	Bacillales	Paenibacillaceae	
B08b	*Paenibacillus etheri*	Site 2	Supernatant	99.8% *Paenibacillus etheri*	Firmicutes	Bacilli	Bacillales	Paenibacillaceae	
B09	*Paenibacillus etheri*	Site 1	Thallus	99.8% *Paenibacillus etheri*	Firmicutes	Bacilli	Bacillales	Paenibacillaceae	
B10	*Paenibacillus etheri*	Site 1	Supernatant	99.8% *Paenibacillus etheri*	Firmicutes	Bacilli	Bacillales	Paenibacillaceae	OL891638
B11	*Paenibacillus etheri*	Site 1	Supernatant	99.8% *Paenibacillus etheri*	Firmicutes	Bacilli	Bacillales	Paenibacillaceae	
B12	*Paenibacillus etheri*	Site 2	Supernatant	99.8% *Paenibacillus etheri*	Firmicutes	Bacilli	Bacillales	Paenibacillaceae	
B13	*Paenibacillus etheri*	Site 2	Thallus	99.8% *Paenibacillus etheri*	Firmicutes	Bacilli	Bacillales	Paenibacillaceae	
B14	*Paenibacillus etheri* ^a^	Site 2	Supernatant		Firmicutes	Bacilli	Bacillales	Paenibacillaceae	
B15	*Paenibacillus etheri* ^a^	Site 2	Supernatant		Firmicutes	Bacilli	Bacillales	Paenibacillaceae	
B16	*Paenibacillus etheri* ^a^	Site 1	Supernatant		Firmicutes	Bacilli	Bacillales	Paenibacillaceae	
B17	*Paenibacillus etheri*	Site 2	Supernatant	99.8% *Paenibacillus etheri*	Firmicutes	Bacilli	Bacillales	Paenibacillaceae	
B23	*Lichenibacterium* sp.^a^	Site 1	Supernatant		Proteobacteria	α‐proteobacteria	Hyphomicrobiales	Lichenibacteriaceae	
B24b	*Lichenibacterium* sp.	Site 1	Thallus	97% *Lichenibacterium ramalinae*	Proteobacteria	α‐proteobacteria	Hyphomicrobiales	Lichenibacteriaceae	OL891639
B24c	*Lichenibacterium* sp.^a^	Site 1	Thallus		Proteobacteria	α‐proteobacteria	Hyphomicrobiales	Lichenibacteriaceae	
B33a	*Microbacterium paraoxydans*	Site 1	Supernatant	99.7% *Microbacterium paraoxydans*	Actinobacteria	Actinomycetia	Micrococcales	Microbacteriaceae	OL891640
B33b	*Microbacterium paraoxydans*	Site 1	Supernatant	99.7% *Microbacterium paraoxydans*	Actinobacteria	Actinomycetia	Micrococcales	Microbacteriaceae	
B34	*Paenibacillus etheri* ^a^	Site 1	Thallus		Firmicutes	Bacilli	Bacillales	Paenibacillaceae	
B35	*Microbacterium paraoxydans* ^a^	Site 1	Supernatant		Actinobacteria	Actinomycetia	Micrococcales	Microbacteriaceae	
B38	*Paenibacillus etheri* ^a^	Site 2	Supernatant		Firmicutes	Bacilli	Bacillales	Paenibacillaceae	
B39	*Caballeronia mineralivorans*	Site 2	Thallus	99.3% *Caballeronia mineralivorans*	Proteobacteria	β‐proteobacteria	Burkholderiales	Burkholderiaceae	OL891641
B46	*Paenibacillus etheri* ^a^	Site 1	Thallus		Firmicutes	Bacilli	Bacillales	Paenibacillaceae	

^a^ Morphological identification.

**TABLE 3:** Microalgae isolated from *R*. *geographicum*

					Classification				
Isolates	BLAST and Phylogenetic identification	Location	Deposit method	BLAST result	Phylum	Class	Order	Family	Accession number
B21	*Apatococcus lobatus*	Site 1	Supernatant	99.6% *Apatococcus lobatus*	Chlorophyta Pascher	Trebouxiophyceae	Chlorellales	Chlorellaceae Brunnthaler	OL891598
B22	*Coccomyxa viridis*	Site 1	Supernatant	100% *Coccomyxa viridis*	Chlorophyta	Trebouxiophyceae			OL891599
B26	*Coccomyxa viridis*	Site 1	Supernatant	100% *Coccomyxa viridis*	Chlorophyta	Trebouxiophyceae			
B40	*Coccomyxa viridis* ^a^	Site 1	Supernatant		Chlorophyta	Trebouxiophyceae			
B41	*Apatococcus lobatus* ^a^	Site 1	Supernatant		Chlorophyta Pascher	Trebouxiophyceae	Chlorellales	Chlorellaceae Brunnthaler	
B44	*Coccomyxa viridis* ^a^	Site 1	Supernatant		Chlorophyta	Trebouxiophyceae			

^a^ Morphological identification.

Sequence similarities to available sequences in the NCBI GenBank database were analysed using the Basic Local Alignment Search Tool (BLASTn program http://blast.ncbi.nlm.nih.gov/Blast.cgi) (Altschul et al., 1990). Fungal ITS reference sequences were selected to carry out a phylogenetic analysis. All sequences were aligned with MAFFT v6.814b (Katoh et al., 2002) using Geneious®6.1.8. The PhyML method (Guindon & Gascuel, 2003) was used via the Geneious platform to generate maximum‐likelihood phylogenetic tree with the following setting: GTR substitution model, 100 bootstraps, estimated transition/transversion ratio, estimated proportion of invariable sites, estimated gamma distribution, branch length and optimized substitution rate. Phylogenetic tree was visualized and edited with megaX (Kumar et al., 2018).

The following criteria were used to determine the taxa from the GenBank database and the phylogenetic tree: for sequence identities >99%, the species were accepted; for sequence identities of 97%–99%, only the genus was accepted. When sequences matched at 100% with several species, only the genus was accepted.

## RESULTS

### Isolation and identification of isolated microbiota

The morphological observation of the fungal and bacterial isolates from *R*. *geographicum* led to the isolation of 68 fungi, 24 bacteria and six microalgae.

**FIGURE 1:**
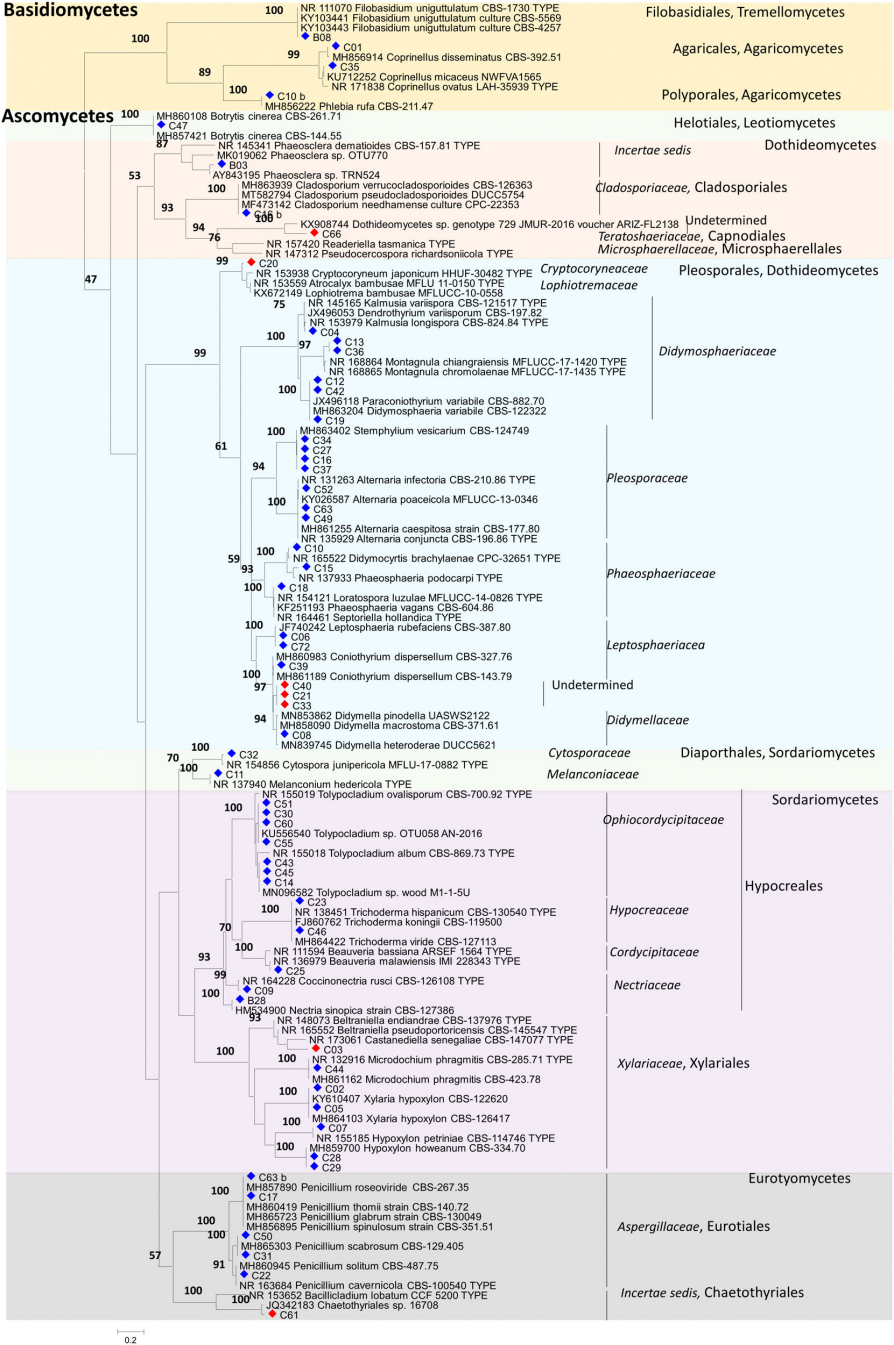
Maximum‐likelihood phylogenetic tree based on samples ITS sequences and closest ITS reference sequences from GenBank. The tree was obtained by applying the PhyML method in the Geneious platform. Bootstrap values >50% are indicated above branches. Sequences generated in this study are indicated with diamonds. Red diamonds correspond to non‐identified isolates

Among the 68 fungal isolates, 62 were identified on molecular basis, distributed in 43 phylogenetic groups (Figure 1) and six were morphologically related (Table 1; Figure 1). Thirty‐seven isolates were identified at the species level and 19 at the genus level. Twelve isolates belonging to nine different phylogenetic groups could not be identified either at the species or the genus level.

Concerning bacteria, among the 24 isolates, 12 were identified at the species level and two isolates at the genus level after comparison with the GenBank database (Table 2). Only one isolate was identified at family level and nine isolates were morphologically related. Out of the six microalgal isolates, three were identified at the species level and three were morphologically linked to each other. (Table 3).

### Diversity of isolated fungal communities

**FIGURE 2:**
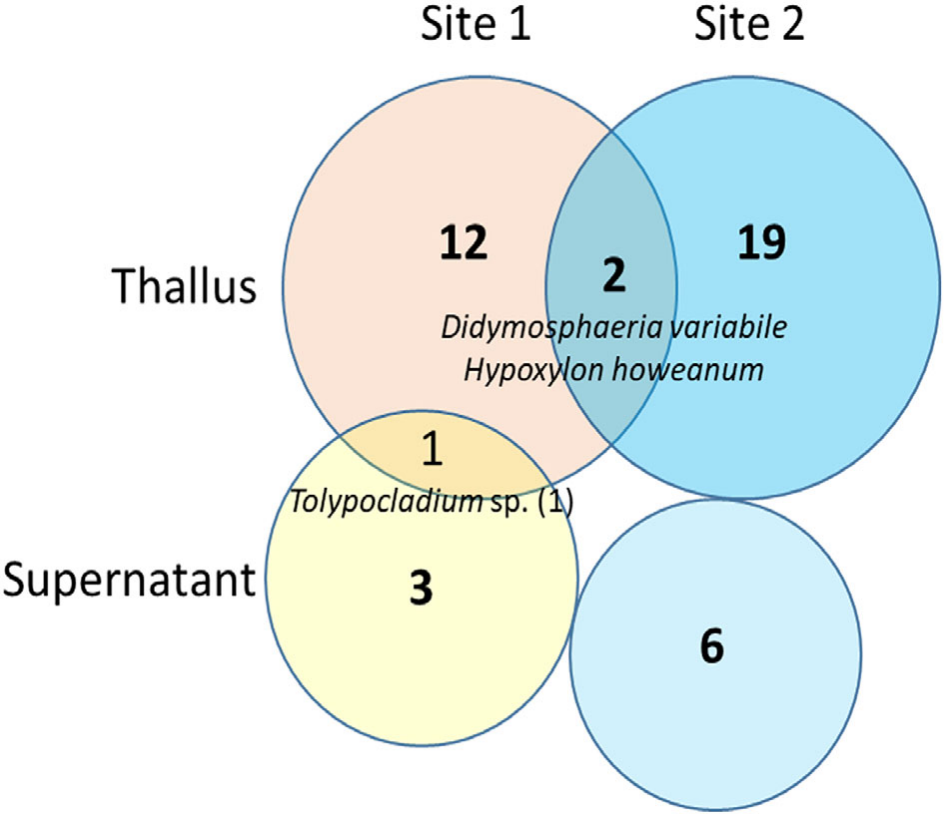
Venn diagram representing the number of shared and exclusive fungi isolated from *R*. *geographicum* comparing the different collect locations and methods of isolation

Ascomycota is the dominant phylum with 62 isolates, depicting 91.2% of the total fungal isolates (Figure 2). They are distributed in four classes, 10 identified and two unidentified orders and 21 families. The most abundant class was Dothideomycetes (32 isolates, 47%) followed by Sordariomycetes (22 isolates, 32.3%) and Eurotiomycetes (six isolates, 8.8%). Leotiomycetes are represented by two isolates. At the order level, Pleosporales (27 isolates, 39.7%) are dominant, followed by Hypocreales (14 isolates, 20.5%) and Xylariales (seven isolates, 10.3%). At the family level, Pleosporaceae (nine isolates, 13.2%) is the most abundant followed by Ophiocordycipitaceae and Didymosphaeriaceae (seven isolates, 10.3% each). Finally, at the species level *Stemphylium vesicarium* (five isolates, 7.3%) is more present.

Basidiomycota phylum is poorly represented with only six isolates corresponding to 8.8% of the total isolates but five different taxa were identified. With two isolates each, Agaricomycetes and Tremellomycetes classes are the most abundant class (33.3%). Only one undetermined isolate represented the Cystobasidiomycetes class.

Comparing the two sampling sites, 25 strains were isolated and identified from site 1 versus 43 isolated and identified from site 2 (Table 1). The two sites share two taxa: *Didymosphaeria variabile* and *Hypoxylon howeanum*. Among the 25 isolates from site 1, four were isolated from the supernatant representing four taxa including two undetermined; 21 were isolated from the thallus representing 14 taxa. *Tolypocladium* sp. (1) is common to the thallus and supernatant deposit methods. Out of the 43 isolates from site 2, seven were isolated from the supernatant representing three taxa including three undetermined; 37 were isolated from the thallus representing 19 taxa including four undetermined (Figure 2). From washing water, only 11 isolates among the 68 isolates were obtained. However, five of them were characterized as undetermined taxa. Moreover, one yeast, *Filobasidium uniguttulatum*, was isolated.

### Diversity of isolated bacterial communities

This work led to the isolation of 24 bacterial strains belonging to five different taxa. Three taxa were recovered from site 1. *Lichenibacterium* sp. and *Microbacterium paraoxydans* were only found at the first site while the second site allowed the isolation of one undetermined taxon. *Paenibacillus etheri* was found at both locations.

While Firmicutes is the dominant phylum (15 isolates, 62.5%), Proteobacteria phylum represents 20.8% of total bacterial isolates and Actinobacteria 16.7%. At the class level, Bacilli (15 isolates, 62.5%) is dominant, followed by Actinomycetia and Alphaproteobacteria (respectively four isolates, 16.7%). At the order level, Bacillales (15 isolates, 62.5%) is in the majority, followed by Micrococcales and Hyphomicrobiales (respectively four isolates, 16.7%). At the family level, the most abundant is Paeanibacilleae (15 isolates, 62.5%), followed *ex aequo* by Microbacteriaceae and Lichenibacteriaceae (respectively four isolates, 16.67%). At the genus level the most abundant bacteria are *Paenibacillus* (15 isolates, 62.5%), followed by *Lichenibacterium* (four isolates each, 16.7%).

### Isolation of microalgae

The long 6 months incubation period permitted the isolation of six microalgae. All of them were isolated from the supernatant and from the site 1. They all belong to the Trebouxiophyceae family and two species were identified including *Apatococcus lobatus* and *Coccomyxa viridis*.

### Selectivity of agar media used

**FIGURE 3:**
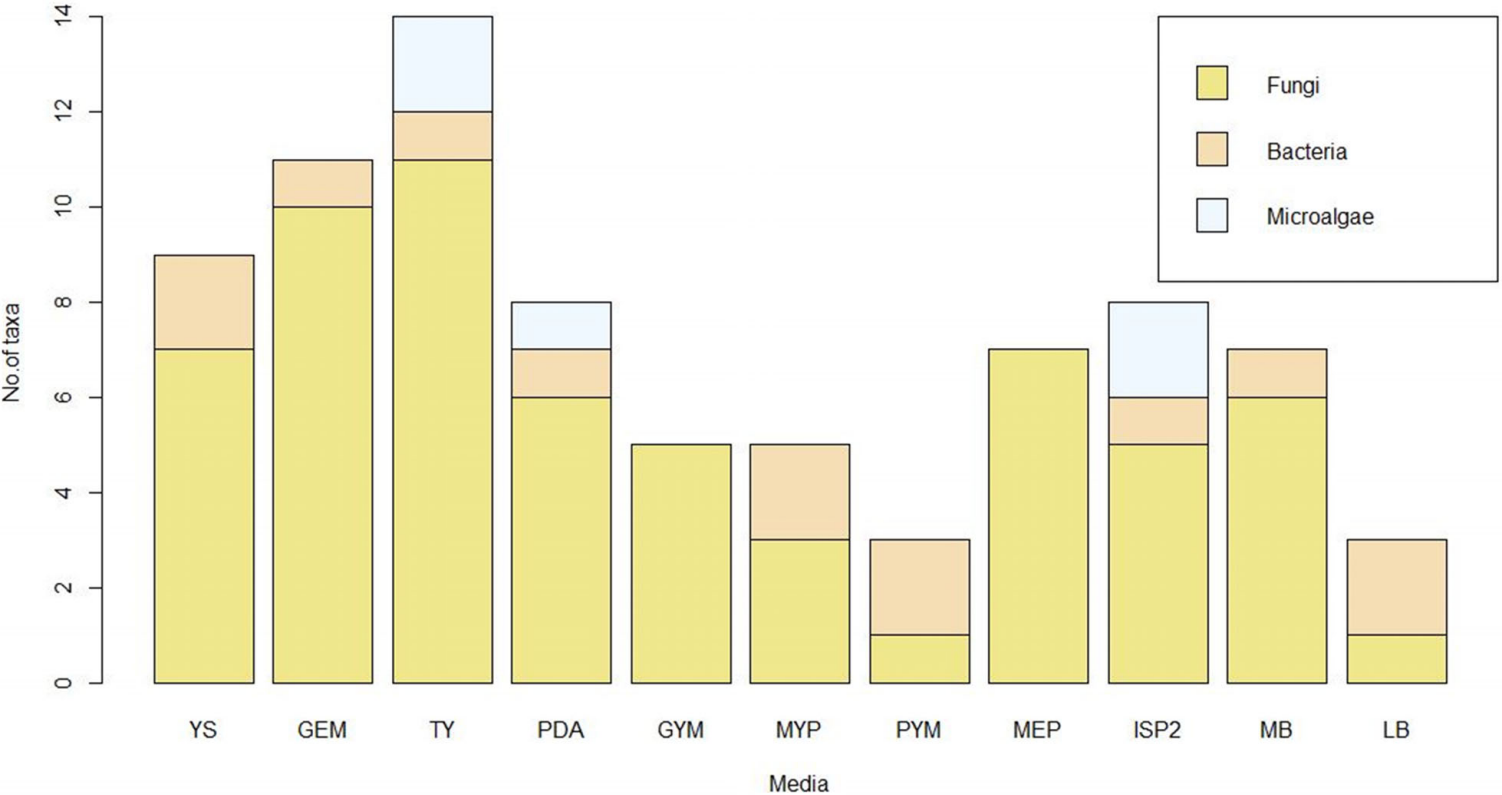
Comparison of the number of taxa identified according to the media used

Originally, the media were chosen for the isolation of bacteria. As no antifungal compound was used, fungi also grew. The lichen mycobiota diversity was higher with the TY medium (25.6%), closely followed by the GEM (23.3%) and the YS and MEP media (16.3%) (Figure 3). With taxa diversity of 14%, the PDA and MB media allowed the growth of six different taxa each. The lowest diversity recovery has been observed for PYM and LB media with only one taxon (2.3%). On LB medium six isolates were purified but five of them could not be further cultivated under axenic conditions. Twelve unidentified fungal strains belonging to nine different phylogenetic groups were mostly recovered on PDA (25%), YS, GYM and MEP (16.7%) media.

Concerning bacteria, YS, MYP, PYM and LB media permitted to recover two different taxa (40%). GYM and MEP media did not allow any bacterial growth contrary to previous isolation (not published).

The six microalgal strains were able to grow as well on PDA, TY, GYM as on ISP2 media (Figure 3).

### Comparison of different deposit methods

**FIGURE 4:**
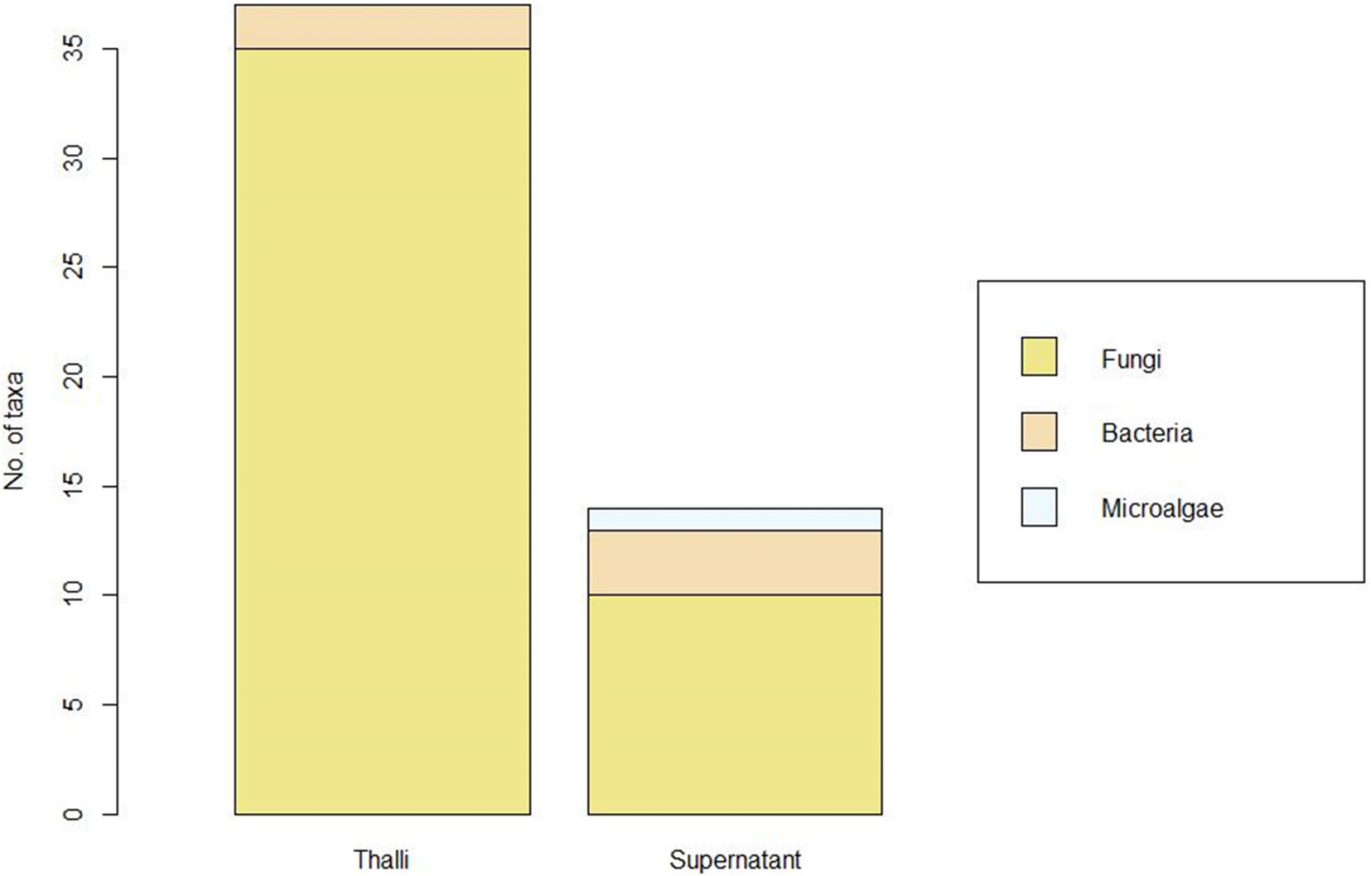
Comparison of the number of fungal, bacterial and microalgal strains identified according to the deposit method used

The deposit method of lichen material onto Petri dishes had an effect on microbial growth (Figure 4). Indeed, for fungi, the suspension spreading led to an isolation of 10 different taxa including five undetermined. Two of the 10 taxa were shared with the thalli pieces deposit. On the other side, the lichen bacteriobiont showed a greater diversity when the suspension was spread. Microalgae were only expanded when the suspension of lichen material was spread plated.

### Comparison of locations

Over the 68 fungal isolates, 28 (41.2%) were recovered from the site 1 and 40 (58.8%) from the site 2. Site 1 presented 16 different taxa including two undetermined while site 2 presented 30 taxa. Sites 1 and 2 had six shared taxa, although some taxa are not shared as *Tolypocladium* sp. which are only found on site 1. Moreover, *Stemphylium vesicarium* and undetermined Pleosporales are only recovered from samples of the second site. Concerning the bacterial isolates, *Paenibacillus etheri* was common to both sites. The undetermined bacteria and *Caballeronia mineralivorans* were isolated from site 2. *Microbacterium paraoxydans* and *Lichenibacterium* sp. were only found from the site 1. Finally, all the microalgae were isolated from the site 1.

## DISCUSSION

The diversity of lichen‐associated microbiota of *R*. *geographicum* was, to our knowledge for the first time, investigated by a culture‐dependent approach. The *R*. *geographicum* lichen samples were chosen because it was poorly studied and the rocky sea coasts represent some of the most extreme habitats for living organisms. Two different spatially proximate sites were chosen: site 1, more inland than the site 2 located on the edge of the cliff.

This work enabled the isolation and molecular identification of 62 fungal isolates, 15 bacterial isolates and three microalgae. Site 2, the closest to the cliff, appeared to be richer than the site 1 with more undetermined taxa from the thallus as well as from the supernatant. Indeed, for 12 fungal isolates belonging to nine different taxa, the genus is not represented in GenBank. These genera have never been sequenced or even described and are therefore non‐studied. While many species disappear every day, this work suggests that coastal and wild environments still have an unrevealed variety to offer and that lichens constitute a great reservoir of microbial diversity which can be recovered by multiplying the culture‐dependent techniques.

Surprisingly, among the Basidiomycota, three Agaricomycetes, which were never previously described as endolichenic, were found from thallus and exclusively from site 2. One of them, *Coprinellus disseminatus* described as a wood decomposer (Singh et al., 2009), does not have much to decay on such a surface. The presence of another taxon *C*. *micaceus*, described as being able to biosorb 100% of lead (Albert et al., 2020), could be explained by a pollution fact: Brest Bay is known for its very high level of Pb due to bombing attacks during the World War II and by the intensive agriculture (Chiffoleau, 2017). Endolichenic fungi are often host‐generalists with regard to the lichens in which they occur. They are more closely related to endophytic symbionts than to saprotrophic fungi, suggesting that their associations with lichen thalli are not purely incidental (Chagnon et al., 2016). As a matter of fact, *C*. *micaceus* could thus play a role of a ‘protector’ as it could have been previously described for some bacterial strains (Cernava et al., 2017).

Most of the fungal isolates (91.2%) identified belong to the Ascomycota phylum, which is consistent with previous results (Lagarde et al., 2018). Among Ascomycota, Dothideomycetes were particularly abundant (44.8%) followed by Sordariomycetes (32.8%). It is known that the growth form of the lichen hosts influences the diversity of the associated fungi. Hence, taxa belonging to Dothideomycetes are mainly isolated from crustose thalli on rocks (Muggia & Grube, 2018). Very few fungal genera found herein have already been reported from lichens. We can cite endolichenic fungi belonging to *Aureobasidium*, *Cladosporium*, *Penicillium*, *Trichoderma* and *Xylaria* (Lagarde et al., 2018). At the species level, it is interesting to note that, only one species described in our study, *Botrytis cinerea*, a known pathogenic plant fungus, was already isolated from an epiphytic lichen *Ramalina fastigiata* (Lagarde et al., 2018). These findings highlight the novel and high diversity of the microflora associated to *R*. *geographicum*.

Regarding the bacterial diversity, while one study reported, using fingerprinting method (DGGE) and clone libraries, the presence of Acidobacteria and α and β‐Proteobacteria from *R*. *geographicum* (Bjelland et al., 2011), Firmicutes was, in our study, the dominant bacteria phylum (15 isolates, 62.5%) only represented by the genus *Paenibacillus* followed by *α*‐Proteobacteria represented by *Lichenibacterium* sp. then by Actinobacteria. These results highlight the importance of using different and complementary methods in order to better describe a microbial community. *Lichenibacterium* species (*L*. *ramalinae* and *L*. *minor*), the second most abundant bacterial species, were reported as β‐carotene‐producing bacteria and were already isolated from subantarctic lichens (Pankratov et al., 2020). Antarctic lichens housed also *C*. *mineralivorans*, an atmospheric nitrogen fixer (Noh et al., 2021). Strains of *Paenibacillus* have been described as being especially common constituents of the lichen‐associated microbiota fraction (Grube & Berg, 2009). In addition, trying to explain the low rate of bacterial isolates and the high percentage of *Paenibacillus* strains identified, we have based ourselves on some studies which previously reported that Firmicutes from lichens were widely considered as producers of antibiotics and enzyme inhibitors (Swamy & Gayathri, 2021) or were mycorrhizal helper bacteria (Poole et al., 2001). At the species level, *P*. *etheri*, the most abundant bacterial species found in this study, was isolated from hydrocarbons polluted soil and has been described as a methyl *tert*‐butyl ether degrader (Guisado et al., 2016). Interestingly, this species was already isolated from an *R*. *geographicum* sample collected 4 years ago on the Brittany Coasts (data not published), converging with the isolation of *C*. *micaceus*. Moreover, *Arthrobacter* sp. and *Microbacterium paraoxydans* have been described as being able of bioremediation (Manzoor et al., 2021; Sayyed et al., 2019) and for the last species as arsenic and lead degrader (Kaushik et al., 2012). The location of lichens harvested, la Pointe de Crozon, was also polluted by the Amoco Cadiz and Erika oil spills in 1974 and 1999 respectively, we can ask ourselves if these bacteria have some kind of ability to improve and help the lichen to live in such an unfriendly environment. This observation could also be supported by the identification of a *Cladosporium* strain from the site 2 which has been described as hydrocarbons tolerant and capable of bioremediation (Birolli et al., 2018; Velez et al., 2020), another ‘protector’ species as described above.

Most of the culture‐dependent studies concentrated on filamentous Ascomycetes, lichen‐inhabiting Basidiomycetes or yeasts have only rarely been isolated (Duarte et al., 2016; Santiago et al., 2015; Zhang et al., 2016). It was shown that metabarcoding using ITS1 and ITS2 permitted the detection of Basidiomycetes but no Cystobasidiomycetes (Banchi et al., 2018; Fernández‐Mendoza et al., 2017). In both studies, the lichens surfaces were not sterilized prior to performing metabarcoding. Hence, as nothing went off the samples, it might explain the infrequency of Basidiomycetes isolation in lichen‐associated microbiota works. In our study, prior to the isolation steps, the lichen material was not commonly sterilized but just washed (Yang et al., 2021). One Cystobasidiomycete strain (site 1) and *Filobasidium uniguttulatum* (site 2) were then identified and isolated from the supernatant spreading deposit method. This supports the hypothesis that Cystobasidiomycetes are epilichenic rather than endolichenic (Černajová & Škaloud, 2019). In 2016, it was suggested that these yeasts may play a role in lichens' phenotype and hypothesized that the yeasts may represent yet another obligatory constituent of the lichen symbiosis (Spribille et al., 2016), hypothesis criticized later (Oberwinkler, 2017). More recently, a positive correlation was made between the abundance of Basidiomycete secondary fungal symbionts in the lichen *Bryoria tortuosa* with the visible production of the specialized metabolite vulpinic acid in the shared extracellular matrix between the core ascomycete symbiont and the yeasts (Tagirdzhanova et al., 2021). With added partners, the complexity of species interactions increases (Mark et al., 2020). As the Basidiomycete yeasts diversity is still poorly known and the opinion diverse, it might be hard to conclude on either one or the other hypothesis but their diversity can be expected to be tremendous.

The crustose lichen *R*. *geographicum* has an unusual biology consisting of discrete areolae containing the Trebouxia algal symbiont, growing on the surface of a non‐lichenized Lecanoromycetes fungal hypothallus. This morphology poses several interesting biological questions, including how lichenization occurs. It seems that lichenization occurs by chance contact between free‐living algae, which is considered as the ‘primary photobiont’ (Voytsekhovich and Beck, 2016), and short‐lived fungal hyphae (Armstrong, 2011). As *R*. *geographicum* colonizes some of the most hostile environments on earth, ‘additional photobionts’ in thalli of trebouxioid lichens, that might have been trapped from the outside and temporarily included in the thallus, could be considered as a possible additional source of nutrients and might be connected with the scarcity of the required alga in the habitat or with the presence of primary photobiont in an insufficient amount for thallus formation (Voytsekhovich and Beck, 2016) supporting the argument of ‘the symbiotic playground of lichen thalli’ (Muggia et al., 2013). Whereas the ‘primary photobiont’, Trebouxia, was not recovered, as no specific methodology for such an isolation was used, ‘additional photobionts’, *C*. *viridis* and *A*. *lobatus*, were isolated. While the genus *Coccomyxa*, which can be lichenicolous algae or lichenized photosynthetic partners in lichens (Malavasi et al., 2016) as described in several studies (Cao et al., 2018; Gustavs et al., 2015), displays a wide variety of lifestyles and some of its strains have been identified as extremotolerant and generalists with low nutritional needs (Gustavs et al., 2015), *Apatococcus* taxon, striking ecological differences with the lichen photobiont (Chrismas et al., 2021; Gustavs et al., 2016), seems to be a specialized and slow‐growing alga (Gustavs et al., 2016). It is interesting to note that a strain of *Coccomyxa viridis* was also isolated from the *R*. *geographicum* sample collected 4 years ago. The isolation of such taxa could be explained as a synergistic association in order to counteract the extreme conditions encountered at the sampling sites; the presence of multiple coexisting photobionts with different physiological properties provides an ecological advantage: marine‐derived photobionts promote survival in the intertidal zone while freshwater‐derived photobionts may allow better photosynthesis during the rain events (Chrismas et al., 2021). Moreover, a recent study related the variation of photobiont diversity depending on the growth stage of the thalli, the geographic location and the habitat. An important point which was also revealed is the risk of overestimation of photobiont diversity from small thalli (Molins et al., 2021). While it has always been possible to assess relationships among the fungal partners through microscopy of their complex structures, the same cannot be said for the algal partner. The tedious culture of lichen photobionts has been the only possibility to distinguish species of microalgae and cyanobacteria isolated from lichen symbioses. The enhancement of molecular methods and development of specific primers for algal gene loci has improved the knowledge of the diversity and variability of photosynthetic partners (Grube and Spribille, 2012). The questions which can be asked are if they are merely epibionts or only distributed in low abundance within the lichens or only spotted in certain parts of the thallus (Guzow‐Krzemińska, 2006; Grube and Muggia, 2010; Casano et al., 2011). A study hypothesized, by analysing both washed and unwashed lichen samples (Muggia et al., 2013), that the epithalline algae communities host numerous algal species, and if not separately considered, might lead to an overestimation of photobiont diversity in lichens in general and a direct improper function in the lichen symbiosis. But as emphasized, on the other hand, it may confer advantages in the lichen's ability to counteract environmental changes or to occupy extreme environments mixing strategies to fine‐tune their association (Casano et al., 2011; Molins et al., 2018). Indeed, a higher variety of symbiotic associations could be helpful when changing environments and therefore might be the rule in lichens living on a wide variety of substrates, as *R*. *geographicum*, and in diverse habitats like the very hostile environment of the Britain Atlantic Coast. They could finally correspond to a habitat‐adapted symbiosis (Rodriguez et al., 2008; Casano et al., 2011) and different partnerships can be tested at low risk for the entire thallus structure (Muggia et al., 2013). Even if the Sanger direct sequencing approach gave clues about fungus–algal association, distribution patterns and diversity in lichens, it also can lead to oversimplify diversity (Voytsekhovich and Beck, 2016), thus, underestimating all the complexity of the symbiotic association and under‐detecting less abundant co‐occurring photobiont partner (Moya et al., 2017). Therefore, the best way for characterization of the algal partner is a combination of culture‐dependent and independent methodologies on freshly collected lichen materials (Thüs et al., 2011).

The isolation and identification of microbial diversity associated with the lichen *R*. *geographicum*, despite the small sampling, from the coastal area of La Pointe de Crozon proved to be very rich and broad. Coastal microbial communities are complex and interact with their surrounding environments (Fuhrman et al., 2015). Thus, the isolates recovered herein could be influenced by saline habitats, e.g. sea sprays and tides. Halotolerant bacteria and fungi are those capable of growing in the absence as well as in the presence of relatively high salt concentrations (Kushner, 1978). Among the isolated strains, *C*. *disseminatus* has been described as halotolerant (Khusnullina et al., 2018). Moreover, among the identified genera, e.g. *Aureobasidium*, *Penicillium*, *Leptosphaeria*, *Tolypocladium*, *Trichoderma*, *Cladosporium*, *Alternaria*, *Paenibacillus*, many include taxa isolated from marine environments (Zalar et al., 2007; Zuccaro et al., 2008; Gal‐Hemed et al., 2011; Khusnullina et al., 2018; Ghafari et al., 2019; Araújo et al., 2020; Gopal and Anandham, 2020). The wide range of metabolic activities of this microbial consortium might be responsible for synergistic effects of multi‐strains microbes to improve lichen fitness, as it was shown for plants (Zahir et al., 2019). Furthermore, the dominant bacteria phylum Firmicutes, only represented by the genus *Paenibacillus*, was described as being an abiotic stress reducer (Cernava et al., 2017). *Paenibacillus glucanolyticus* sp., a halotolerant strain, was isolated from marine environment (Ghafari et al., 2019). Moreover, the abundance of *P*. *etheri* within our samples could play a special role in counteracting environmental stress such as salinity by becoming active and playing key ecosystem roles in response to system perturbations.

Finally, we can mention that the deposit method used impacts the microbial isolation. Indeed, while fungi were mostly isolated from thalli, bacterial isolates and microalgae were better recovered from supernatant. Comparing the fungal recovery rates of the suspension plate spreading deposit method and the thalli deposit method, the latter allowed us to isolate and identify 87.5% of the total lichen mycobiota. This could be explained by the fact that all lichen thalli host a community of cryptic fungi and that many lichenicolous fungi are endothallic, i.e. form their mycelium inside the thallus (Arnold et al., 2009; Muggia et al., 2014, 2016, 2017; Muggia & Grube, 2018; U'Ren et al., 2010). Moreover, even if the size of the lichen thalli deposited could have appeared to be very small, the diversity of the fungi recovered was great. Indeed, as previously described, the isolated density and diversity are inversely related to the size of the thallus (Yang et al., 2021).

## CONFLICT OF INTEREST

All authors declare no conflict of interest.

## Data Availability

Data available on request from the authors.
